# Annotations

**Published:** 1896-06-06

**Authors:** 


### Annotations.

### Cat's Paws at the Chelsea Hospital for Women.

A very curious resolution was passed by the governors of the Chelsea Hospital for Women, at a special meeting held immediately after the last general meeting, on May 20th. By it power was given to the weekly board to suspend any member of the honorary medical staff for not longer than a month, and to a meeting of the council, summoned for the purpose, to dismiss him altogether; and those who know the history of this indifferently-managed institution may well inquire what is the meaning of this new move. Is it done to promote the good work of the hospital and the benefit of the patients, or is it another of those petty personal manœuvres for which this hospital has become so celebrated? During recent years many changes have taken place in the staff, and some, even, in that most adhesive body the board of management; but through all these vicissitudes certain members of the old board and the old staff have always come up again. And the more clear it becomes that there is an inner ring within the ostensible managing bodies, and that in this inner ring there is a strong medical influence, the more suspiciously people will look upon this new power which they have claimed of dismissing from the staff all who make themselves obnoxious to the powers that be. It has been pointed out before, and we need not hesitate to point out again, how dangerous to the welfare of a hospital may be the preponderating influence over the board which is apt to be gained by any member of the staff who is willing to give largely and collect largely for the benefit of the institution. Infinitely more dangerous, however, does the influence of wealth become when, by such powers of suspension and dismissal as we have described, the board may be induced to act as cat's paw for the removal of opposition and the suppression of criticism.

### Unnecessary Inquests.

Some little time ago, in commenting on a pamphlet by Mr. Braxton Hicks, the moral of which was "In cases of doubt come to your coroner," we pointed out that if that advice were acted on up to the letter, the work of the coroner's office would be enormously increased. Perhaps we ought to have gone further and said that unless the coroner were to exercise considerable judgment the work of juries would be so increased as to become an intolerable burden. We are constantly hearing the cry of the oppressed juryman at being called upon to break his work and sacrifice his time in the holding of unnecessary inquests. On May 28th an inquest was held at the Southwark coroner's court on a woman who died suddenly a week after her confinement, she having been attended by a student, and the jury chose to express much indignation at the authorities of Guy's Hospital allowing such cases to be attended by unqualified men, their idea being that if the doctor had been qualified he could have certified the cause of death. As a fact, however, the death was a sudden and unexpected one; and would have had to be reported to the coroner in any case. The error was the holding of an inquest at all. If all doubtful cases are to be reported to the coroner, he ought to hold some form of investigation before calling a jury together. In this case, if there was cause for suspicion, why was there no *post-mortem* examination? Either a *post-mortem* was necessary, or an inquest was unnecessary. The fact is, it was unnecessary, and we cannot but imagine, so often does this sort of thing come up, that there is some influence besides the discovery of the cause of death that leads to the holding of inquests. We believe that some small fees accrue to some of the minor officials engaged every time an inquest is held, and it is to be feared that the unnecessary inquests, the holding of which so often brings discredit on the whole system of the coroner's court, are often really due to a search for pickings on the part of those in office.

### The Late Sir Russell Reynolds.

A blameless man, a man of high aims and of excellent life, has passed away in Sir Russell Reynolds, president of the Royal College of Physicians. It is not given to every man to possess an outstanding personality and to amaze the world. If a man takes the powers he possesses and makes of them an entirely worthy use throughout the whole of life his contemporaries say of him that he has done and deserved well. To the class of excellent well-doers Sir Russell Reynolds belonged. In early life Dr. Reynolds devoted his powers to the study and elucidation of the structure and functions of the nervous system. To us it seems almost incredible that reflex action in the nervous system should ever have presented any obstacles to the inquiring mind. But, as a matter of fact, in the days before Sir Charles Bell, Marshall Hall, and others, the functions of the nervous system appeared to be locked up in a casket of impenetrable mystery, a casket for which there was no key. The key once found, the mystery was unravelled; but this very fulness of present knowledge and simplicity of demonstrable theory is all to the honour of such men as Reynolds, who leave us to reap where they sowed. The medical profession is both enriched and purified in character and capacity by the lives of such men as Reynolds. Not destitute of ambition, not unmoved by the prevailing hunger of the times for notoriety and wealth, they set out in early life on a course of honest well-doing, and resolve that if such a course may lead to distinction, the distinction shall be welcome; but if otherwise, they will be content to remain obscure. Not always in medicine does this course lead to fame, as witness the life and history of the great and truly venerated Bristowe. In Reynold's case the honours came, and when they came they were worn with modest dignity as they had been won by honourable work. Sir Russell Reynolds has died prematurely—he was but sixty-eight years of age. He has not died too soon for fame; but his friends cannot help a feeling of regret that a youth of stern and unremitting labour, followed by a manhood of almost more exacting anxiety and toil, should not have had at least a few years of quiet and dignified repose before the grave claimed for eternal silence a writer and teacher who had deserved so entirely the regard of his friends and of the world.

